# Genome-Wide DNA Methylation Analysis of Mammary Gland Tissues From Chinese Holstein Cows With *Staphylococcus aureus* Induced Mastitis

**DOI:** 10.3389/fgene.2020.550515

**Published:** 2020-10-19

**Authors:** Mengqi Wang, Yan Liang, Eveline M. Ibeagha-Awemu, Mingxun Li, Huimin Zhang, Zhi Chen, Yujia Sun, Niel A. Karrow, Zhangping Yang, Yongjiang Mao

**Affiliations:** ^1^Key Laboratory for Animal Genetics, Breeding, Reproduction and Molecular Design of Jiangsu Province, College of Animal Science and Technology, Yangzhou University, Yangzhou, China; ^2^Joint International Research Laboratory of Agriculture and Agri-Product Safety, The Ministry of Education of China, Yangzhou University, Yangzhou, China; ^3^Agriculture and Agri-Food Canada, Sherbrooke Research and Development Centre, Sherbrooke, QC, Canada; ^4^Centre for Genetic Improvement of Livestock, Department of Animal Biosciences, University of Guelph, Guelph, ON, Canada

**Keywords:** genome-wide DNA methylation, mastitis, *Staphylococcus aureus*, mammary gland tissue, Chinese Holstein

## Abstract

*Staphylococcus aureus* intramammary infection is one of the most common causes of chronic mastitis in dairy cows, whose development may be associated with epigenetic changes in the expression of important host defense genes. This study aimed to construct a genome-wide DNA methylation profile of the mammary gland of Chinese Holstein cows (*n* = 3) following experimentally induced *S. aureus* mastitis, and to explore the potential gene regulatory mechanisms affected by DNA methylation during *S. aureus* mastitis. DNA was extracted from *S. aureus*-positive (*n* = 3) and *S. aureu*s-negative (*n* = 3) mammary gland quarters and subjected to methylation-dependent restriction-site associated DNA sequencing (Methyl-RAD Seq). Results showed that C^m^CGG/C^m^CWGG DNA methylation sites were unevenly distributed and concentrated on chromosomes 5, 11, and 19, and within intergenic regions and intron regions of genes. Compared with healthy control quarters, 9,181 significantly differentially methylated (DM) C^m^CGG sites and 1,790 DM C^m^CWGG sites were found in the *S. aureus*-positive quarters (*P* < 0.05, |log2FC| > 1). Furthermore, 363 C^m^CGG differently methylated genes (DMGs) and 301 C^m^CWGG DMGs (adjusted *P* < 0.05, |log2FC| > 1) were identified. Gene ontology and KEGG enrichment analysis indicated that C^m^CGG DMGs are involved in immune response pathways, while the C^m^CWGG DMGs were mainly enriched in gene ontology terms related to metabolism. The mRNAs of 526 differentially methylated C^m^CGG genes and 124 differentially methylated C^m^CWGG genes were also significantly differentially expressed (RNA-Seq data) in the same samples, herein denoted differentially methylated and expressed genes (DMEGs) (*P* < 0.05). Functional enrichment analysis of DMEGs revealed roles related to biological processes, especially the regulation of immune response to diseases. C^m^CGG DMEGs like *IL6R*, *TNF*, *BTK*, *IL1R*2, and *TNFSF8* enriched in several immune-related GO terms and pathways indicated their important roles in host immune response and their potential as candidate genes for *S. aureus* mastitis. These results suggest potential regulatory roles for DNA methylation in bovine mammary gland processes during *S. aureus* mastitis and serves as a reference for future epigenetic regulation and mechanistic studies.

## Introduction

Mastitis, which is defined as inflammation of the mammary gland, is one of the most common production-limiting diseases of the dairy industry (Beniæ et al., 2018). Mastitis not only has significant negative impacts on milk production, including decreased milk yield and milk quality (Detilleux et al., 2015), but also influences the reproductive system (Kumar et al., 2017) as well as increases susceptibility to other diseases (Cha et al., 2013). Mastitis is caused by varied pathogens leading to the development of subclinical/chronic (25–65% incidence worldwide) or clinical (∼5% incidence worldwide) infections (Dufour et al., 2012). *Staphylococcus aureus* (*S. aureus*), a Gram positive bacterium, is the major cause of chronic mastitis and accounts for up to 64% of total mastitis cases in Canada (Reksen et al., 2006). *S. aureus* mastitis is usually asymptomatic, persistent, easily reoccurs and some *S. aureus* isolates have developed antibiotic resistance (Makovec and Ruegg, 2003; Jensen et al., 2013). Preventive measures to control the occurrence and spread of *S. aureus* IMI (intramammary infections) are hampered by false-positive bacterial diagnostic tests, sudden outbreaks of IMI and poor therapeutic response to antibiotic treatment (Sampimon et al., 2009).

Mastitis results from the interaction between environment (pathogens) and genetic factors. Attempts to control mastitis through improved farm environment and management practices, and application of antibiotics have not eradicated the disease. The possibility to use genetic selection to improve mastitis resistance was born when heritable genetic variation in the incidence of mastitis was demonstrated (Lush, 1950). However, the use of conventional breeding strategies based on phenotypic selection to eradicate mastitis have shown limited success due to the low heritability of the trait (Emanuelson et al., 1988; Carlén et al., 2004; Koivula et al., 2005; Vallimont et al., 2009). Genomic selection has recently been identified as a complementary or alternative tool for selection to reduce mastitis (Martin et al., 2018; Weigel and Shook, 2018). To support genomic selection, several genome wide association studies (GWAS) have identified genes, markers and quantitative trait loci (QTL) significantly associated with mastitis (Wang X. et al., 2015; Suravajhala and Benso, 2017; Cai et al., 2018; Welderufael et al., 2018; Kurz et al., 2019).

In recent years, attention has been devoted to the study of transcriptome and post-transcriptional regulatory mechanisms of *S. aureus* mastitis, with aim to find additional biomarkers from different perspectives, for use in genomic selection, molecular diagnostics and for therapeutic purposes (Wang et al., 2016; Luoreng et al., 2018; Han, 2019). Furthermore, growing evidence demonstrates that epigenetics also profoundly influence health, growth, development and production traits of cattle (Ibeagha-Awemu and Zhao, 2015; Reynolds et al., 2017; Sun et al., 2019). Several studies have reported the potential contribution of epigenetic mechanisms like DNA methylation (Song et al., 2016; Zhang et al., 2018), histone modification (He et al., 2016) and non-coding RNA (Li et al., 2015; Pu et al., 2017; Ma et al., 2019) to mastitis risk. As the most characterized epigenetic regulatory mechanism, DNA methylation was reported to regulate mammary gland health (Zhang et al., 2018; Chen et al., 2019; Wang et al., 2019). For example, hypermethylation of *CD4* gene promoter was reported to repress its expression during clinical mastitis (Wang et al., 2013; Usman et al., 2016). Genome-wide DNA methylation analysis of peripheral blood lymphocytes identified 1,078 differentially methylated genes between cows with *S. aureus*-mastitis and healthy cows, as well as the potential of *NRG1*, *MST1*, and *NAT9* genes to serve as biomarkers of *S. aureus* mastitis progression (Song et al., 2016). Furthermore, DNA methylation was found to regulate the expression of bovine immune-related genes, including *IL6*, *IL8*, and *TLR4*, in response to lipopolysaccharide-induced mastitis (Korkmaz, 2018). Additionally, abnormal expression of *IL6* was revealed to be due to altered DNA methylation rather than genetic mutation during clinical mastitis (Zhang et al., 2018). Moreover, methylation changes in *NCKAP5* and transposon MTD were associated with the development of *S. aureus* mastitis (Wang et al., 2020), while hypomethylation of global DNA methylation induced by *S. aureus* infection repressed DNA methyltransferase activity in bovine mammary epithelial cells (Wu et al., 2020).

DNA methylation occurs mainly in the form of CpG (addition of methyl group to the fifth position of cytosine base followed by guanosine), and to a lesser extend at non-CpG sites, including CpA, CpT, and CpC. However, the functions of non-CpG sites are not yet elucidated or still controversial (Patil et al., 2014). Methods used for whole genome DNA methylation profiling include whole genome sequencing approaches (e.g., whole genome bisulfite sequencing) and enrichment based immunoprecipitation or enzyme based methods that reduce genome complexity through enzymatic digestion followed by bisulfite treatment and next generation sequencing (Li et al., 2010). Methyl-RAD Seq is one of such enzymatic based methods that uses methylation-dependent restriction enzymes (FspEI, MspJI, etc.) to reduce genome size and focus the analysis on methylation rich regions (Wang S. et al., 2015). Methyl-RAD Seq has been improved in technological ways making it more advantageous than other enzymatic based methods such as reduced representation bisulfite sequencing (RRBS) (Gu et al., 2011), MeDIP-seq (Taiwo et al., 2012), MethylCap-seq (Brinkman et al., 2010) and MethylSeq (Brunner et al., 2009). C^m^CGG and C^m^CWGG (W = T or A) are two primary target sites of methylation-dependent restriction enzymes used for some DNA methylation sequencing approaches including Methyl-RAD Seq, providing the data for next-step functional analysis for both CpG and non-CpG sites (Wang S. et al., 2015).

The reports above support the hypothesis that DNA methylation contributes to susceptibility to, or outcome of *S. aureus* mastitis. However, the global DNA methylation pattern of mammary gland tissue from dairy cows infected with *S. aureus* mastitis is ambiguous, and related functional reports are scarce. To further test this hypothesis, the aim of the present study was to profile the genome-wide DNA methylation landscape of mammary gland tissue of Holstein cows, and to identify differentially methylated sites and genes and their potential involvement in the host response to *S. aureus*-induced mastitis. Data from this study is expected to provide reference for further functional studies and mechanistic exploration of epigenetic mechanisms underlying *S. aureus* mastitis and to contribute to improve management of *S. aureus* infection in dairy herd.

## Materials and Methods

### Animals and *S. aureus* Challenge

Three primiparous Holstein cows in mid-lactation (142 ± 25 days) and without a history of disease were selected from Yangzhou University Dairy Farm (Jiangsu, China) for this study. Cows were in their first parity and were under routine farm management and feeding regime. The milk somatic cell counts (SCC) of cows was monitored for 3 weeks prior to the experimental IMI challenge. Furthermore, milk samples were collected weekly and cultured at 37°C for 24 h on Aureus ChromoSelect Agar Base Media (HiMedia, Mumbai, India) to detect the presence of *S. aureus*. Only cows negative for *S. aureus* and having a SCC of ≤100,000 cells/mL were used (Rainard et al., 2018). The *S. aureus* strain Rosenbach ATCC^®^29213^TM^ (ATCC, Manassas, VA, United States) was used for the IMI challenge as described previously (Li et al., 2015). Briefly, 5 mL *S. aureus* suspension (1 × 10^7^ CFU, SA treatment) was injected into one mammary gland quarter of each of the three cows, while the same volume of aseptic vehicle phosphate buffer (PBS) solution was injected into another quarter of each of the same three cows (CL treatment). Milk samples were aseptically collected from each quarter just before *S. aureus* IMI challenge and then at 6, 12, 18, and 24 h post IMI challenge. Quantification of SCC and test for the presence of *S. aureus* in milk samples were done as described previously with three experimental repeats for each sample from each sampling time (Li et al., 2015). Mammary gland tissues were surgically collected at 24 h post IMI challenge from each quarter and immediately sectioned into small pieces (around 50 mg) and snap frozen and stored in liquid nitrogen. All the above animal experiments followed the guide of animal welfare and use procedures of Yangzhou University and permission to carry out the study was approved by the Institutional Animal Care and Use Committee of Yangzhou University (NO: 201404018).

### DNA Isolation

DNA was extracted from three *S. aureus* positive mammary tissues (SA treatment) and three control tissues (CL treatment) using QIAamp DNA mini kit (Qiagen Inc., Shanghai, China) according to manufacturer’s protocol. The concentration and purification of DNA samples were measured with a NanoDrop ND-1000 (NanoDrop Technologies, Wilmington, DE, United States) and confirmed by agarose gel.

### DNA Methylation Library Construction and Sequencing

Preparation of genome-wide DNA methylation sequencing libraries was carried out according to method reported earlier (Wang S. et al., 2015) using Methyl-RAD Seq method. Briefly, DNA was digested with 4 U FspEI (NEB) at 37°C for 4 h followed by adaptor ligation and PCR amplification. Barcodes were introduced through PCR for multiplex sequencing. The sequences of primers, adaptors and barcodes are listed in Supplementary Table S1A. The PCR products were purified using the QIAquick PCR Purification Kit (Qiagen Inc., Shanghai, China), and single-end sequenced (1 × 36 bp) on an Illumina HiSeq 2500 system (Illumina, San Diego, United States). Sequencing was performed to achieve a coverage depth of 10x.

### DNA Methylation Sequencing Data Analysis

The raw sequences were trimmed with Trimmomatic (v 0.36) (Bolger et al., 2014) to remove adaptor sequences. Reads with more than 8% ambiguous bases, or more than 15% bases with a quality score of less than 30 were also trimmed. Clean reads that passed quality control and with expected restriction sites were defined as enzyme reads and were used for subsequent analyses. Sequences containing C^m^CGG or C^m^CWGG (W = T or A) (methylation on second C) with FspEI restriction sites were extracted from the bovine reference genome UMD 3.1.1 (GCF_000003055.6 *Bos Taurus* UMD 3.1.1) and used as reference for methyl-RAD data analysis. Enzyme reads were mapped to the reference sequences with SOAP (v2.21) (Li et al., 2008) allowing two mismatches (-M 4 -v 2 -r 0). The methylation sites that were found in all SA and/or CL samples and with >3× coverage were retained for further analyses. The normalized read-depth (reads per million, RPM) was used as the unit for relative quantification of level of DNA methylation. SnpEff (v4.3p) (Cingolani et al., 2012) was used to obtain information on untranslated regions (UTR) from the bovine reference genome (UMD 3.1.1). The distribution of mapped enzyme reads to different genic regions {3′ UTR, 5′ UTR, Upstream [2,000 bp upstream of transcription start site (TSS)], exon, intron and downstream [2,000 bp downstream of transcription termination site (TTS)]} and intergenic regions was achieved with bedtools (v 2.25.0) (Quinlan and Hall, 2010).

### Identification of Differently Methylated Genes and Functional Enrichment Analysis

The methylation level of a gene was represented by the sum of the sequencing reads of all its methylated sites. Differential methylated gene (DMG) analysis was accomplished with EdgeR (v 3.26.4) (Robinson et al., 2010). DMGs meeting the thresholds of adjusted *P*-value < 0.05 and |log2FC| > 1 were considered significant. Significantly DMGs were subjected to gene ontology (GO) and Kyoto Encyclopedia of Genes and Genomes (KEGG) pathways functional enrichment analysis with the ClusterProfile (v 3.8) program in R package (Yu et al., 2012).

### Association Analysis of Differently Methylated Genes and Transcriptome Data

The DMGs (adjusted *P-*value < 0.05) were filtered against the mRNA transcriptome data of the same samples (Liao, 2014; Chen et al., 2019, b) to select the differentially methylated genes that were also transcriptionally differentially- expressed (adjusted *P-*value < 0.05); these will subsequently be referred to as differentially methylated and expressed genes (DMEG). The DMEGs were used for GO and KEGG pathway enrichment with the ClusterProfiler package in R software, and network visualization was performed with ClueGO^1^ (Bindea et al., 2009).

### Validation of DNA Methylation by Bisulfite Sequencing PCR (BSP)

To validate the reliability of Methyl-RAD Seq data, bisulfite sequencing PCR (BSP) was used to identify the methylation level of three DMEGs (*CXCR1*, *CDH13*, and *METTL13*). The Methyl primer express (v1.0) was used to design the PCR primers (Supplementary Table S1B). Genomic DNA (around 1,000 ng) from each tissue sample was treated with bisulfite sodium using EpiTect Plus Bisulfite kit (Qiagen, Shanghai, China). The BSP reaction was performed in 20 μL, including 1 μL (10 ng) of bisulfite-treated DNA, 1 μL of forward and reverse primer (10 μmoL each), 10 μL of 2 × T5 super PCR MIX (Qingke, Beijing, China) and 7 μL of RNase-free water. The PCR program was as follows: 94°C for 5 min; 40 cycles of 94°C for 5 s, 58°C for 30 s and 72°C for 30 s; and 72°C for 10 min. The PCR product size and purity were confirmed by agarose gel electrophoresis and then cloned into the pMD19-T vector (Shenggong, Shanghai, China). About 20 positive clones for each gene and sample were randomly selected for sequencing (Shenggong, Shanghai, China). The BiO_Analyzer^2^ was used to analyze the sequence results.

### Statistical Analyses

The DNA methylation levels of C^m^CGG and C^m^CWGG sites between SA and CL groups were compared with Fisher’s exact Test implemented in EdgeR (v 3.26.4) program. The enrichment score in the gene ontology (GO) analysis in ClusterProfile (v 3.8) was calculated with the following formula:

Enrichment score=mnMN

Where N represents the number of all genes with GO annotation, *n* is the number of DMGs with GO annotation. M and *m* are the number of all genes and DMGs annotated in a specific GO term, respectively. Hypergeometric contribution test was used to calculate the significance of gene enrichment in each GO term, and adjusted *P*-value < 0.05 was considered significant.

## Results

### Induction of Mastitis in Chinese Holstein Cows With *S. aureus*

**FIGURE 1:**
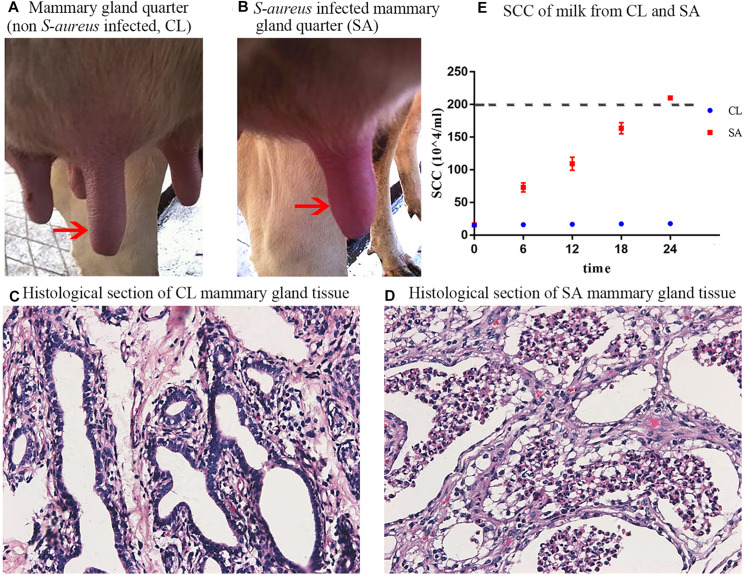
Clinical observation and diagnosis of *S. aureus*-induced mastitis. **(A)** Healthy mammary gland quarter; **(B)** Mammary gland quarter with clinical symptoms: red, swollen and indurated, 24 h after *S. aureus* induction; **(C)** Histological section of healthy mammary gland quarter; **(D)** Histological section of *S. aureus*-infected mammary gland quarter, showing atrophic acinar wall and damaged acinar structure with lots of magazines gathered in the acini; **(E)** Individual SCC of milk collected from the healthy (CL group: quarters A1, B1, and C1) and *S. aureus*-infected (SA group: quarters A2, B2, and C2) mammary gland quarters during the 24 h post *S. aureus* induction. The red arrows shows the control **(A)** and challenged **(B)** mammary gland quarters.

As compared with the CL treatment (Figure 1A), the infected mammary gland quarters of the SA treatment showed significant symptoms of inflammation (redness, swelling and warmness to the touch) at 24 h after *S. aureus* challenge (Figure 1B). Furthermore, cows of the SA treatment showed avoidance behavior and refused to be milked when the infected mammary gland quarter was touched. Histological sections of the infected mammary gland quarters showed atrophic acinar wall and damaged acinar structure with lots of impurities gathered in the acini, that could be exfoliated mammary epithelial cells and inflammatory cells (Figure 1D) as compared to uninfected quarters (Figure 1C).

Compared with CL, the milk yield of SA treatment decreased, with flocculent precipitate and increased SCC (*P* < 0.05) (Figure 1E), indicative of mastitis. Bacterial culture revealed that milk samples collected from all the mammary gland quarters before IMI challenge were *S. aureus* negative, whereas after IMI, *S. aureus* was cultured from the SA treatments but not in the CL treatments. In summary, these results confirmed successful induction of *S. aureus*-induced mastitis in the challenged quarters and a healthy state in control quarters.

### Genome-Wide DNA Methylation Profile of Mammary Gland Tissue

**FIGURE 2:**
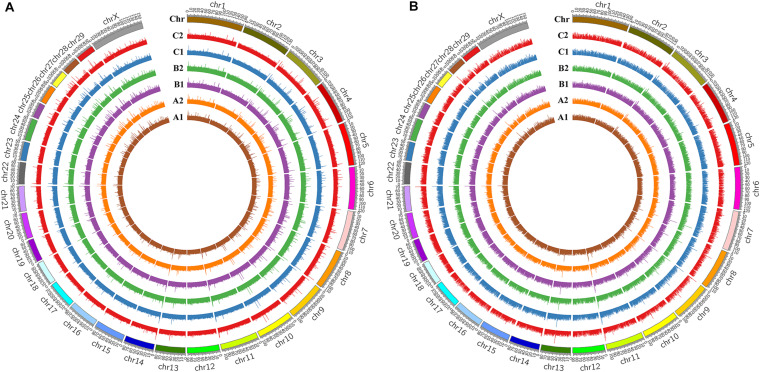
DNA methylation profiles of mammary gland tissues from Chinese Holstein cows. **(A)** C^m^CGG methylation map; **(B)** C^m^CWGG methylation map (W = A or T); C^m^CGG means the methylation whose primary target site is C^m^CGG, while C^m^CWGG means the methylation with C^m^CWGG site (W = A/T/H); A1, B1, and C1 indicate the whole genome wide DNA methylation patterns of healthy mammary gland quarters, while A2, B2, and C2 indicate quarters with *S. aureus*-induced mastitis.

The level of genome-wide DNA methylation was analyzed by Methyl-RAD Seq. A total of 81,125,685 raw reads were generated from the six samples, with an average of 13,520,947 raw reads per sample (Table 1). Reads that mapped to multiple positions (mapping rates of 67.7 to 70.13% of clean reads) were removed, only uniquely mapped reads (mapping rates of 26.93 to 29.09%) were kept for further analysis. The C^m^CGG and C^m^CWGG (W = A or T) methylation maps were generated to show the genome-wide DNA methylation distribution in the studied samples (Figure 2 and Supplementary Table S2). The C^m^CGG and C^m^CWGG methylation sites were unevenly distributed across bovine chromosomes (BTA); BTA with the most C^m^CGG methylation sites were BTA 11, 5, and 19, while C^m^CWGG methylation sites were most distributed on BTA 5, 11, and 1. Additionally, C^m^CGG and C^m^CWGG methylation sites were more concentrated at the BTA ends.

**TABLE 1:** Generated reads and mapping statistics of Methyl-RAD Seq sequence data from *Staphylococcus aureus* induced and healthy udder tissues of Chinese Holstein cows.

Samples	Raw reads	Clean reads	Error rate	Enzyme reads	Q20	Q30	Multi mapped reads	Multi-mapping rate	Unique mapped reads	Unique mapping rate
A1	13479949	13467408	0.99907	12910973	99.87%	99.67%	9053897	70.13%	3857076	29.87%
B1	13724935	13711869	0.999048	13251576	99.88%	99.70%	9151743	69.06%	4099833	30.94%
C1	13411585	13398863	0.999051	12483616	99.88%	99.70%	8464402	67.80%	4019214	32.20%
A2	14104057	14090675	0.999051	13650097	99.88%	99.69%	9381653	68.73%	4268444	31.27%
B2	13165243	13152778	0.999053	12812360	99.88%	99.69%	8932597	69.72%	3879763	30.28%
C2	13239916	13226976	0.999023	12399152	99.88%	99.69%	8394779	67.70%	4004373	32.30%

*A1, B1, and C1 represents healthy udder tissues of Chinese Holstein cows (CL group); A2, B2, and C2 represents *S. aureus* mastitis infected udder tissues of Chinese Holstein cows (SA group); Error Rate is the ratio of clean reads to raw reads; Enzyme Reads represent clean reads containing the expected restriction site and that passed quality control. The multi/unique-mapping rate is based on enzyme reads.*

**FIGURE 3:**
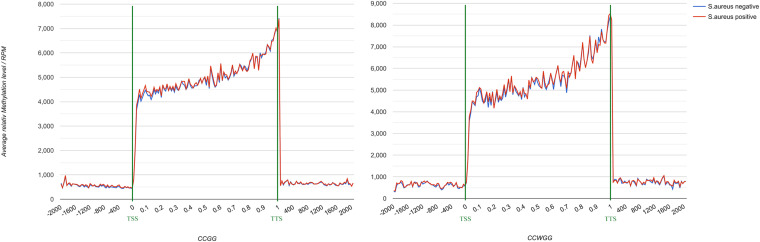
DNA methylation level across gene in mammary gland quarters from Chinese Holstein cows. TSS, transcriptional start site; TTS, transcriptional termination site; Abscissa represents the gene body, expressed as the percentage of gene length (from 0 to 1),the region of 2000 bp before TSS (from –2000 bp to the start of gene body marked as 0), and 2000 bp after TTS [following the end of gene body (marked as 1) until 2000 bp]. Ordinate represents the average relative DNA methylation level (RPM, reads per million) and different colors means different groups.

**FIGURE 4:**
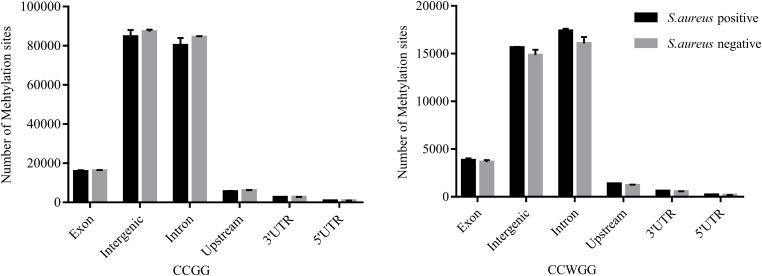
The distribution of DNA methylation sites in functional gene elements. Abscissa represents functional gene elements, and upstream represents 2000 bp region before transcriptional start site. Ordinate represents the number of DNA methylation sites.

To further understand the distribution of DNA methylation sites in the studied mammary gland tissues, gene body, upstream and downstream of genes were analyzed. As shown in Figure 3, the methylation level in gene bodies was higher than in the regions before the TSS and after the TTS and a sharp increase was observed around the TSS and a decrease around the TTS. Moreover, the distribution of DNA methylation sites in different functional gene elements showed enrichment in intergenic regions and introns, followed by exons and upstream regions, while the 3′UTRs and 5′UTRs had less methylation sites (Figure 4).

### DNA Methylation Variations Between *S. aureus*-Infected and Non-infected Mammary Gland Quarters

**FIGURE 5:**
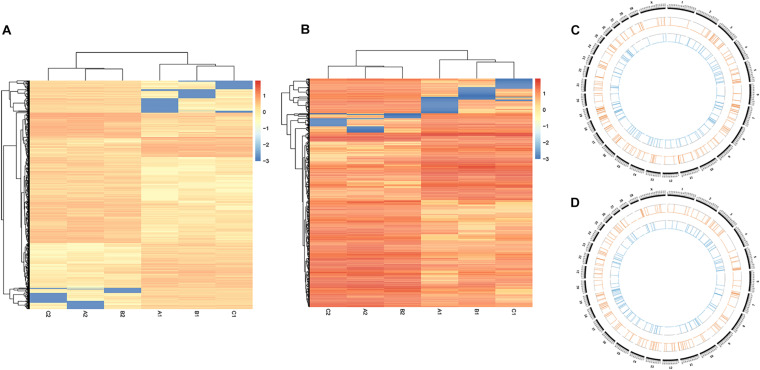
Cluster analysis of DNA methylation levels in healthy and *S. aureus*-induced mammary gland tissues. **(A,B)** are the heat maps for C^m^CGG and C^m^CWGG methylation, respectively. The colors and intensity indicate the methylation level in the heat map. For example, highly methylated sites are in red color and hypomethylated sites are in blue color. **(C)** The distribution of C^m^CGG differently methylated genes across chromosomes, while **(D)** shows the distribution of C^m^CWGG differently methylated genes. Blue lines in the inner circle represent genes with significantly down-regulated methylation levels, while the orange lines in the next circle represent genes with significantly up-regulated methylation levels.

Exploration of the DNA methylation variations between *S. aureus* mastitis and healthy control tissues, showed significant C^m^CGG/C^m^CWGG methylation differences between the two groups (Figures 5A,B). A total of 9,181 C^m^CGG methylation sites and 1,790 C^m^CWGG methylation sites were differentially methylated between SA group and CL group (Supplementary Table S3). Furthermore, differentially methylated sites were unevenly distributed among chromosomes, with higher density of sites on BTA 5, 11, and 19 (Supplementary Table S4). All the methylation sites were annotated to genes to further assess the DNA methylation variations. A total of 363 C^m^CGG DMGs and 301 C^m^CWGG DMGs were obtained (Supplementary Table S5), including 28 DMGs common to the two DNA methylation types, which showed enrichment on BTA 5, 7, 11, 18, and 19 (Figures 5C,D and Supplementary Table S4).

### GO and KEGG Pathway Enrichment Analysis of DMGs

**FIGURE 6:**
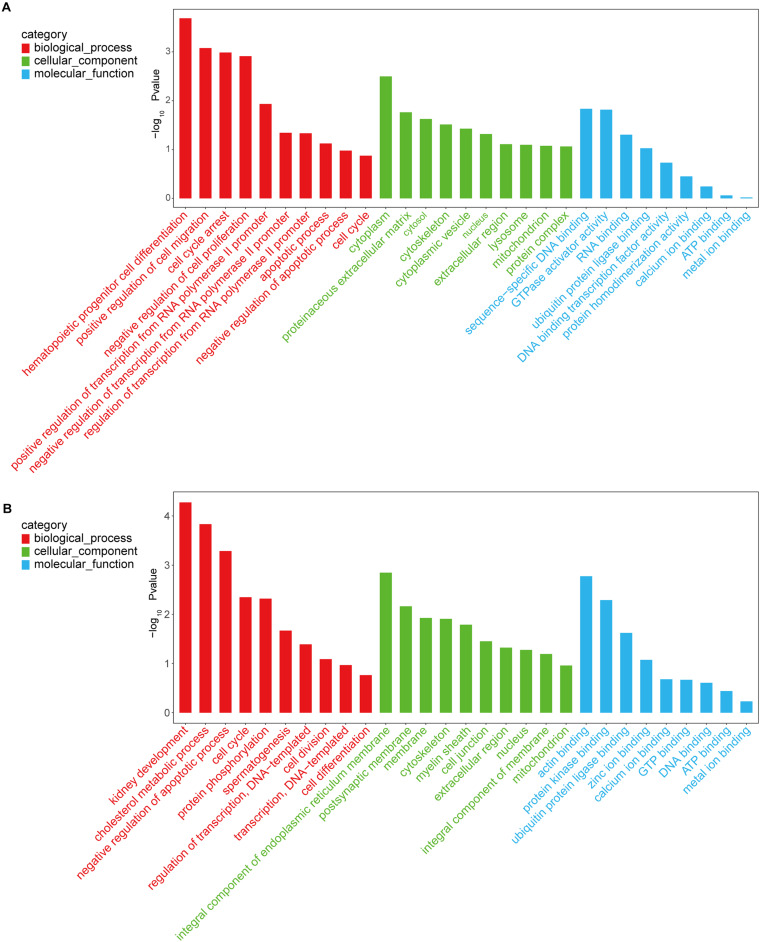
Histogram of gene ontology terms enriched by differently methylated genes. **(A)** Histogram of C^m^CGG differently methylated genes; **(B)** Histogram of C^m^CWGG differently methylated genes. Ordinate represents the adjusted *P*-value (-log_10_ *P*-value). Abscissa represents the GO terms. Only the top 10 GO terms of each category are listed in ascending order of adjusted *P-*value.

Investigation of the potential functions of DMGs through GO and KEGG pathways enrichment analyses revealed that 580 GO terms were significantly enriched by C^m^CGG DMGs, while C^m^CWGG DMGs were significantly enriched in 460 GO terms (adjusted *P*-value < 0.05, Supplementary Table S6). For the C^m^CGG DMGs, the most significant biological process (BP), cellular component (CC) and molecular function (MF) GO terms were “hematopoietic progenitor cell differentiation” (GO:0002244), and “cytoplasm” (GO:0005737) and “sequence-specific DNA binding,” respectively (Figure 6A). “Kidney development” (GO:0001822), “integral component of endoplasmic reticulum membrane” (GO:0030176) and “actin binding” (GO:0003779) were the most significant BP, CC and MF GO terms enriched by C^m^CWGG DMGs, respectively (Figure 6B).

**FIGURE 7:**
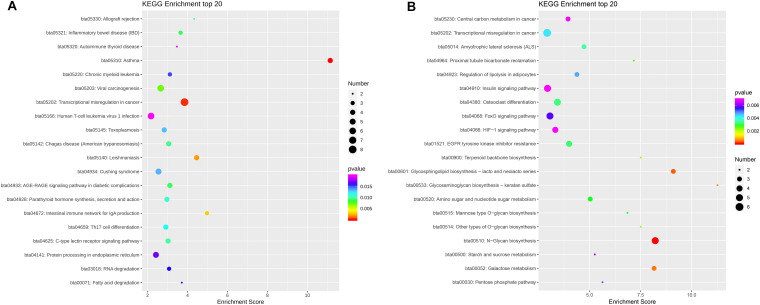
Scatter plot of KEGG pathways enriched by differently methylated genes. **(A)** Scatter plot of C^m^CGG differently methylated genes; **(B)** Scatter plot of C^m^CWGG differently methylated genes. Ordinate represents the significantly enriched KEGG pathways. The abscissa represents the corresponding enrichment score. The color of the spot indicates the adjusted *P-*value of each pathways, while the size of the spot indicates the number of differently methylated genes enriched in that pathway. A bigger enrichment score means better enrichment. Only the top 20 KEGG pathways are listed in ascending order of adjusted *P*-value.

KEGG pathways enrichment analysis identified 40 pathways for the C^m^CGG DMGs and 64 pathways for the C^m^CWGG DMGs (adjusted *P*-value < 0.05) (Supplementary Table S7). The top 20 significantly enriched pathways in each category are shown in Figure 7. KEGG pathways enriched by C^m^CGG DMGs were mainly related to diseases and the immune response. The most significantly enriched pathways for C^m^CGG DMGs were “asthma” (bta05310), “transcriptional mis-regulation in cancer” (bta05202) and “leishmaniasis” (bta05140). Meanwhile, the C^m^CWGG DMGs enriched KEGG pathways were more related to biosynthesis and metabolism. “N-Glycan biosynthesis” (bta00510), “glycosphingolipid biosynthesis” (bta00601) and “galactose metabolism” (bta00052) were the top most significantly enriched pathways for C^m^CWGG DMGs.

### Co-expression Analysis Between DEGs and DMGs to Identify DMEGs

**FIGURE 8:**
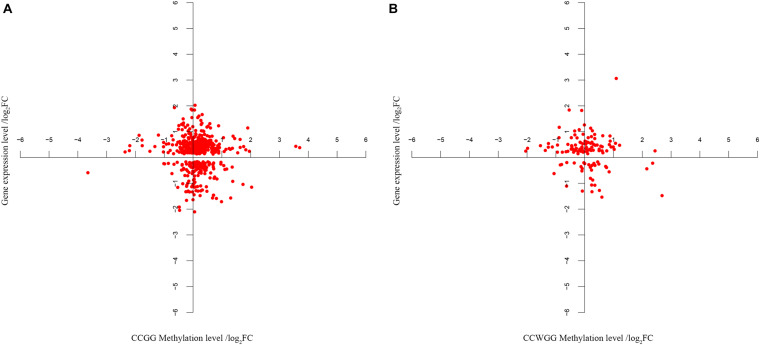
**(A,B)** The co-expression analysis of gene expression and DNA methylation level for DMEGs. Each point in the figure represents one DMEG, the differently methylated gene whose gene expression level is also significantly different in the same tissues. Abscissa indicates the C^m^CGG or C^m^CWGG methylation level, represented by adjusted fold change (log_2_FC). Ordinate indicates the gene expression level (log_2_FC).

**FIGURE 9:**
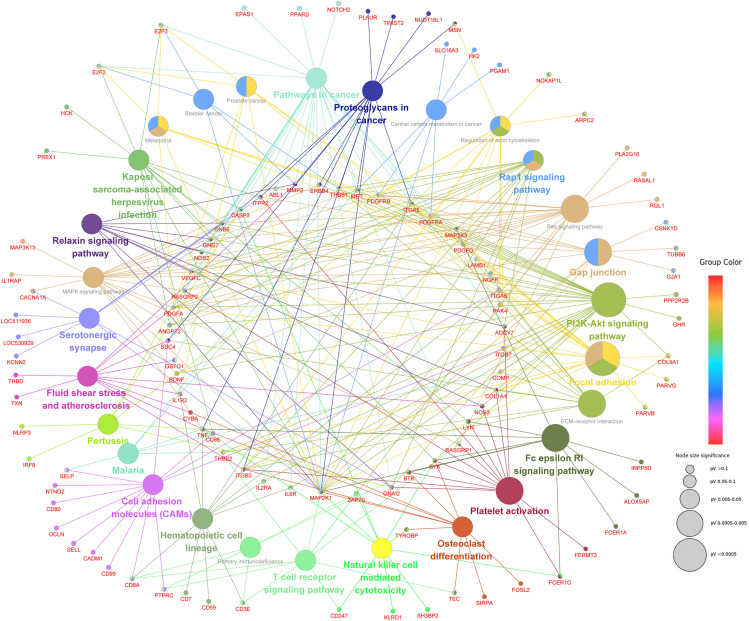
The network of KEGG pathways significantly enriched by C^m^CGG DMEGs. Each node represents a KEGG pathway or a C^m^CGG DMEG enriched in the pathway. The color of the node indicates the cluster of KEGG pathways, that more similar colors mean closer clusters with more similar functions. The size of node indicates the adjusted *P*-value of the corresponding pathway, that a bigger size of the node means more significant enrichment.

The next step was to identify DMEGs, this was achieved by cross-comparing the above DMGs with those that were also previously found to be differentially transcriptionally expressed (Liao, 2014; Chen et al., 2019, b). As shown in Figure 8, 526 C^m^CGG and 124 C^m^CWGG DMEGs were identified. Functional enrichment of the C^m^CWGG DMEGs indicated that only two GO terms [endochondral bone growth (GO:0003416) and calcium ion transmembrane import into cytosol (GO:0097553)] were significantly enriched by seven DMEGs. There were 430 C^m^CGG DMEGs that were significantly enriched in 480 GO terms (adjusted *P* < 0.05, Supplementary Table S8A). The top three most significantly enriched GO terms were “cell adhesion” (GO:0007155), “cell activation” (GO:0001775) and “leukocyte cell-cell adhesion” (GO:0007159). Several enriched (adjusted *P* < 0.05) GO terms were related to cell proliferation, differentiation, migration and adhesion of immune-related cells, such as T cell, lymphocyte and leukocyte, etc (Table 2 and Supplementary Table S8A). Furthermore, GO terms related to the immune response and regulation processes were also enriched by C^m^CGG DMEGs, including “regulation of immune system process” (GO:0002682), “myeloid cell activation involved in immune response” (GO:0002275), etc. Some C^m^CGG DMEGs such as *BTK*, *TNF*, *TNFAIP8L2*, *IL6R*, and *IL18R1*, were enriched in multiple immune processes related GO terms (Supplementary Table S9). Meanwhile, 44 KEGG pathways were significantly enriched (adjusted *P* < 0.05) for C^m^CGG DMEGs. “Focal adhesion” (KEGG:04510) was the most significantly enriched pathway (adjusted *P* = 2.52 × 10^–5^) (Figure 9 and Supplementary Table S8B). The “T cell receptor signaling pathway” (KEGG:04660), “primary immunodeficiency” (KEGG:05340) and other pathways related to cancers and other diseases were enriched (adjusted *P* < 0.05). A total of 102 immune-related C^m^CGG DMEGs enriched in 82 GO terms and 3 KEGG pathways related to immune response, including *IL6R*, *TNF*, *BTK*, *IL1R2*, and *TNFSF8*, are listed in Supplementary Tables S9A,B.

**TABLE 2:** Top 30 significantly enriched gene ontology terms for C^m^CGG DMEGs.

ID	Term	Category	Term *P*-value	Adjusted Term *P*-value
GO:0007155	Cell adhesion	BP	3.70E−15	2.76E−12
GO:0001775	Cell activation	BP	1.05E−11	3.91E−09
GO:0007159	Leukocyte cell-cell adhesion	BP	1.10E−10	2.73E−08
GO:0098609	Cell-cell adhesion	BP	2.31E−10	4.31E−08
GO:0009611	Response to wounding	BP	3.12E−10	4.67E−08
GO:0045321	Leukocyte activation	BP	7.64E−10	9.52E−08
GO:0032879	Regulation of localization	BP	1.15E−09	1.22E−07
GO:0046649	Lymphocyte activation	BP	1.32E−09	1.23E−07
GO:0050865	Regulation of cell activation	BP	3.43E−09	2.85E−07
GO:0042060	Wound healing	BP	8.47E−09	6.33E−07
GO:0030155	Regulation of cell adhesion	BP	1.02E−08	6.94E−07
GO:0009986	Cell surface	CC	1.23E−08	7.66E−07
GO:0042110	T cell activation	BP	1.74E−08	9.98E−07
GO:0016477	Cell migration	BP	3.09E−08	1.65E−06
GO:0048856	Anatomical structure development	BP	5.26E−08	2.62E−06
GO:0022407	Regulation of cell-cell adhesion	BP	6.63E−08	3.09E−06
GO:0030334	Regulation of cell migration	BP	1.19E−07	5.25E−06
GO:0002694	Regulation of leukocyte activation	BP	1.48E−07	6.13E−06
GO:0048870	Cell motility	BP	1.69E−07	6.32E−06
GO:0043062	Extracellular structure organization	BP	1.68E−07	6.62E−06
GO:0030198	Extracellular matrix organization	BP	2.75E−07	9.79E−06
GO:0007596	Blood coagulation	BP	4.63E−07	1.57E−05
GO:0048869	cellular developmental process	BP	4.97E−07	1.61E−05
GO:1903037	Regulation of leukocyte cell-cell adhesion	BP	7.92E−07	2.27E−05
GO:0002682	Regulation of immune system process	BP	9.08E−07	2.51E−05
GO:0051239	Regulation of multicellular organismal process	BP	1.20E−06	3.10E−05
GO:0031012	Extracellular matrix	CC	1.19E−06	3.17E−05

*DMEGs, the C^*m*^CGG differently methylated genes with significantly different gene expression level in the same animal model; BP, biological process; CC, cellular component.*

### Validation of Methyl-RAD Seq Data by BSP

**FIGURE 10:**
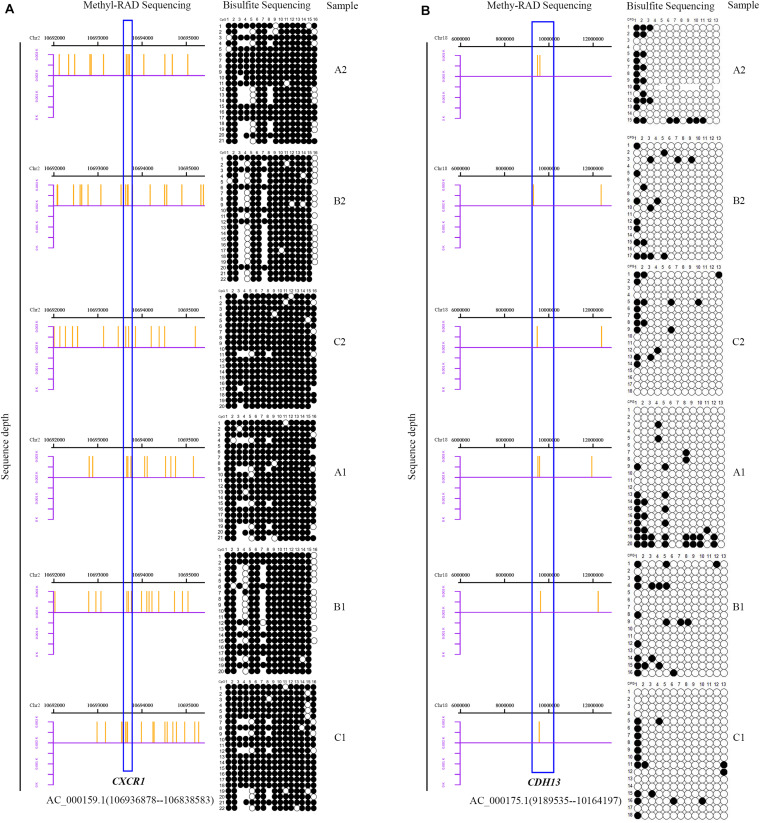
Bisulfite sequencing PCR results. Columns represent the CpG sites and rows represent the clones. Black and white points represent methylated and unmethylated CpG sites, respectively. The blank region indicate that the CpG site was not detected by BSP. The distribution of sequencing reads detected by Methyl-RAD Seq for two genes in the mammary gland quarters of SA group (A2, B2, and C2) and CL group (A1, B1, and C1) are to the right of each figure. The blue boxes represent two genes, *CXCR1* [**(A)**, high methylation level] and *CDH13* [**(B)**, low methylation level].

To assess the reliability and accuracy of the Methyl-RAD Seq data, the methylation levels in promoter regions of three candidate genes were validated by BSP in the SA and CL treatments. *CXCR1*, *CDH13*, and *METTL13* with high, low and zero methylation levels, respectively, were selected. In general, the BSP results were in good agreement with the Methyl-RAD Seq results. For example, the *CXCR1* gene showed high methylation levels (Figure 10A), and the *CDH13* gene showed low methylation levels (Figure 10B), while the *METTL13* gene was almost completely unmethylated (Supplementary Figure S1) according to both methods.

## Discussion

Experimental induction of mastitis by inoculating *S. aureus* into mammary gland quarters has been used to study the transcriptional and post-transcriptional regulatory immune response mechanisms during IMI (Fang et al., 2016; Enger et al., 2019). This strategy was used in this study whereby one mammary gland quarter was inoculated with *S. aureus* to induce mastitis (SA treatment), while one quarter of the same animal was inoculated with PBS and served as the control (CL treatment). Typical clinical symptoms of mastitis were observed in the quarters inoculated with *S. aureus*, such as swelling, redness, warm feeling to the touch, incomplete anatomical structure of acini with exfoliated epithelial cells, as well as significantly increased SCC (>2 million cells/mL) as compared to the healthy PBS quarters, indicating that induction of *S. aureus* mastitis in the SA treatment was successful.

### Global DNA Methylation Pattern of Mammary Gland Tissues Revealed by Methyl-RAD Seq

Methyl-RAD Seq is an improved enzymatic based genome-wide DNA methylation detection method when compared with similar techniques (RRBS, MeDIP Seq, and MethyCap Seq) (Brunner et al., 2009; Taiwo et al., 2012; Huang et al., 2013; Wang S. et al., 2015). Firstly, the process of Methyl-RAD Seq eliminates some enzymatic treatments and purification steps inherent in other methods (RRBS, MeDIP Seq, and MethyCap Seq), which allows application to a wide-range of samples due to it very low DNA input acquirement (about 1 ng). Particularly, the gel purification after DNA digestion is omitted by Methyl-RAD technique, thereby avoiding the denaturation of short fragments (23 or 24 bp) with low melting point in the process of gel electrophoresis (Sanderson et al., 2014). Furthermore, the Methyl-RAD Seq technique is easier and faster, and can be done in a 96-well PCR plate in just 2 days, thus making it an ideal protocol for large-scale methylation detection of numerous samples. Additionally, Methyl-RAD Seq is flexible and users can adjust the label density to meet their specific requirements. Moreover, Methyl-RAD Seq has high specificity, sensitivity and reproducibility with unique abilities to perform qualitative DNA methylation analysis (Huang et al., 2013).

In this study, Methyl-RAD Seq was used for genome-wide DNA methylation profiling of mammary gland tissues of Chinese Holstein cows. Both C^m^CGG and C^m^CWGG methylation sites were unevenly distributed among chromosomes with higher density on BTA 5 and 11 in the mammary gland tissues. A previous study also reported that *S. aureus*-positive cattle had significantly more methylation sites on BTA 11 than *S. aureus*-negative controls (Song et al., 2016), implying a potential regulatory role of DNA methylation in response to *S. aureus* mastitis. We observed the intergenic and intron regions had more methylation sites (adjusted *P* < 0.05) compared with other functional gene elements, which is in agreement with previous studies in other cattle breeds (Song et al., 2016; Fang et al., 2017; Chen et al., 2019, a). Similar to results obtained with other mammalian species (Gim et al., 2015; Song et al., 2016; Zhang X. et al., 2017; Zhang Y. et al., 2017), the methylation level was significantly higher in the gene body than its up- and down-stream regions.

### Potential Functions of Differentially Methylated Genes

DNA methylation plays an important regulatory role in gene expression, which is also influenced by environmental factors and age (Horvath, 2013; Kanzleiter et al., 2015). It has been noted that knowledge on the spatial and temporal regulation of DNA methylation is necessary for understanding the biology of disease (Bergman and Cedar, 2013; Shin et al., 2015). A total of 9,181 C^m^CGG and 1,790 C^m^CWGG methylation sites were identified as differently methylated sites in this study, and were annotated to 363 C^m^CGG and 301 C^m^CWGG DMGs (adjusted *P-*value < 0.05 and |log2FC| > 1). The DNA methylation data in this study was compared with the mRNA transcriptome data generated from these same tissues (Liao, 2014; Chen et al., 2019, b) to explore the effects of DNA methylation on gene expression in response to *S. aureus*-mastitis. We found that a total of 526 C^m^CGG and 124 C^m^CWGG DMGs in this study were also reported as significantly differentially expressed in response to *S. aureus*-induced mastitis (adjusted *P-*value < 0.05) (Chen et al., 2019, 2019b), suggesting that DNA methylation may be one of the regulatory mechanisms of gene expression during *S. aureus* IMI.

DNA methylation of some immune-related genes has been associated with mastitis, such as *CXCR1* and *CDH13*. *CXCR1* gene polymorphism was shown to impact milk production and sensitivity to mastitis, and was selected as a candidate marker for improving bovine resistance to mastitis (Verbeke et al., 2015; Pokorska et al., 2016; Siebert et al., 2017). In addition, CpG sites in the promoter region of bovine *CXCR1* were found to potentially affect its gene expression during *S. aureus*-induced mastitis (Mao et al., 2015). Consistent with Mao et al. (2015) the *CXCR1* gene DNA methylation level in this study was higher in the CL treatment than in the SA treatment. This is supported by its upregulated expression in the SA treatment (Liao, 2014), suggesting an important role in response to *S. aureus* mastitis. In contrast, *CDH13* was reported to have important regulatory roles in human cancers based on a significant correlation of DNA methylation in the *CDH13* promoter with the occurrence, development and prognosis of disease (Yang et al., 2016; Ye et al., 2017; Li Y. et al., 2018). Furthermore, the level DNA methylation in the promoter of *CDH13* is often high during cancer processes (Xu et al., 2016; Wang et al., 2018). In this study, the level of DNA methylation in *CDH13* was higher in SA treatment than in the CL treatment, suggesting a potential regulatory role during *S. aureus* mastitis.

In addition to *CXCR1* and *CDH13*, DNA methylation levels of other immune-related genes, including *IL6R*, *IL10*, *IL17*, and *NFKBIA*, were also significantly different between SA and CL treatments. Moreover, 102 C^m^CGG DMEGs, including *IL6R*, *TNF*, *BTK*, *IL1R*2, and *TNFSF8* were enriched in several immune-related GO terms, indicating their important roles in host immune response, and their potential as candidate genes for improving resistance to *S. aureus* mastitis. A higher level of DNA methylation in *IL6R* was also previously found in mastitis-infected cows; and it was found to regulate the expression of *IL6R* in part by promoting the inclusion of its alternatively spliced exon 2 (Zhang et al., 2018). In contrast, hypomethylation of *IL10* has been reported to promote the incidence of diseases, including *Escherichia coli*-induced mastitis (Modak et al., 2012) and systemic lupus erythematosus (Lin et al., 2012).

The GO functional enrichment revealed the potential roles of C^m^CWGG DMGs in biological processes related to cholesterol metabolic process and protein phosphorylation, as well as molecular functions related to protein kinase binding and ubiquitin ligase binding (Figure 8B). Meanwhile, C^m^CGG DMGs were enriched in several cellular biological processes including cell proliferation, cell differentiation, cell migration, etc., (Figure 8A), suggesting their potential regulatory roles in cell development and diverse biological processes. The C^m^CGG DMGs were significantly enriched in KEGG pathways related to infectious diseases, including Leishmaniasis, Toxoplasmosis and American trypanosomiasis (Figure 9). *S. aureus* mastitis is a common infectious disease of dairy cows, and enrichment of these disease pathways in this study suggests regulatory roles of C^m^CGG DMGs in the host response to *S. aureus*-induced mastitis. Furthermore, C^m^CGG DMGs were also enriched in KEGG pathways related to the immune response, such as “Intestinal immune network for IgA production,” “Th17 cell differentiation,” and “C-type lectin receptor signaling pathway.” C-type lectin is important for the regulation of innate immune and inflammatory processes (Del Fresno et al., 2018), and is known to influence host antimicrobial immunity (Dambuza and Brown, 2015). Th17 cell differentiation plays an important role in immune regulation of T cell during specific immune processes (Sordillo, 2011). It was previously reported that Th17 cells showed specific differentiation in *S. aureus*-mastitis (Zhao et al., 2015), and the diseases and immune-related KEGG pathways in the present study further support the importance of Th17 cells in the host immune response to *S. aureus* mastitis and the regulatory roles of C^m^CGG DMGs.

Interestingly, several GO terms and KEGG pathways related to cell adhesion were enriched by C^m^CGG DMEGs, indicating the important impacts of C^m^CGG methylation on the process of cell adhesion. Cell adhesion has been noted to influence immune cell function impacting cell proliferation, differentiation and migration as well as host-pathogen interaction (Samanta and Almo, 2015; Li J. et al., 2018). The C^m^CGG DMEGs were enriched in GO terms related to inflammatory response, immune system development, regulation of immune response as well as other immune response-regulating signaling pathways indicating their potential regulatory roles in response to *S. aureus*-infection. In addition, we found that C^m^CGG DMEGs were significantly enriched in pathways related to activities of immune cells, such as leukocyte, lymphocyte and T cell, which are important effector cells of the immune system, thus suggesting important roles for these methylated genes in the host immune response to *S. aureus*-mastitis. Lymphocytes are important members of the acquired immune system (Hoekstra et al., 2015) and lymphocyte proliferation and viability are detrimentally affected by *S. aureus* (Nonnecke and Harp, 1988; Hu et al., 2001). T cells activated by monocyte-derived dendritic cells play key roles in immune response to *S. aureus* (Bharathan, 2016). He et al. (2016) also reported that differently expressed genes due to subclinical *S. aureus* mastitis in cattle are involved in the T cell receptor-signaling pathway. These reports are supported by the enriched GO terms related to the immune response regulation and immune system, and KEGG disease and immune related pathways observed in this study.

### Study Limitations and Further Research Prospect

This study constructed the landscape of genome wide DNA methylation of healthy and *S. aureus* infected mammary gland tissues of Holstein dairy cows, providing basic functional prediction of altered methylation sites or genes in relation to *S. aureus* mastitis. However, the relatively small sample size (*n* = 3) limits the reliability of the data. But a major advantage of the data is the fact that infected and health mammary gland quarters where from the same cows which provided room for valid comparisons of methylated sites and also eliminated the bias due to different genetic constitution of infected and control cows if a different set of challenged and control cows were used. Although the sequencing technology (Methyl-RAD) employed for this study had many advantages, such as higher sensitivity and accuracy, the reliability and reference value, but is still limited because of the short-read output, which normally limits the success of unique mapping. This study has provided a general overview and functional prediction of DNA methylation alterations due to *S. aureus* bacteria but further in-depth analyses are needed. For example, the methylation distribution and alteration at the promotor regions, which has significant impacts on gene expression, deserves more detailed analysis. Furthermore, functional analysis and validation of DMGs should be expand and deepen. More cows and a more reliable sequencing method such as whole genome bisulfite sequencing is expected to further enhance the reliability and reference value of detected methylation sites due to *S. aureus* bacteria.

## Conclusion

The genome-wide DNA methylation profile of mammary gland tissues of Chinese Holstein cows with *S. aureus*-induced mastitis was generated by the method of Methyl-RAD Seq, indicating the significantly difference of DNA methylation level between *S. aureus*-challenged and non-challenged quarters. A total of 9,181 C^m^CGG sites and 1,790 DM C^m^CWGG sites were significantly DM between SA and CL treatments. Furthermore, potential functions of 363 C^m^CGG DMGs and 301 C^m^CWGG DMGs were related to the regulation of immune response and metabolism during *S. aureus*-infection, respectively. The functional enrichment of 526 C^m^CGG DMEGs further revealed the regulatory roles of altered methylation of 102 immune-related C^m^CGG DMEGs involved in immune processes and regulation in response to *S. aureus* infection, suggesting their potential as candidate genes for *S. aureus* mastitis. Therefore, these results show that DNA methylation has potential regulatory roles in bovine mammary gland processes during *S. aureus* mastitis and also serves as a reference for future mechanistic studies aiming to better understand the epigenetic regulation of host response to *S. aureus*-induced mastitis.

## Data Availability Statement

Our sequence data have submitted to NCBI (https://dataview.ncbi.nlm.nih.gov/?search=SUB7329711) and accession number is SUB7329711.

## Ethics Statement

The animal use protocol was reviewed and approved by the animal use and care committee of Yangzhou University.

## Author Contributions

MW conducted the experiments, analyzed the data, and drafted the manuscript. ZY and YM designed the experiments and guided the experimental implementation. YL and YS handled the samples and prepared libraries for sequencing. ML, HZ, and ZC were involved in data processing and visualization. EI-A provided inputs in data interpretation and critically reviewed the initial manuscript draft. NK provided inputs in the discussion. All authors reviewed and approved the final draft.

## Conflict of Interest

The authors declare that the research was conducted in the absence of any commercial or financial relationships that could be construed as a potential conflict of interest.

## Footnotes

1 http://apps.cytoscape.org/apps/cluego

2 https://biq-analyzer.bioinf.mpi-inf.mpg.de/index.php
