# Supplementary material for: Genome-Wide DNA Methylation Analysis of Mammary Gland Tissues From Chinese Holstein Cows With Staphylococcus aureus Induced Mastitis
Source: Front Genet. 2020 Oct 19;11:550515. doi: 10.3389/fgene.2020.550515 (PMC7604493; doi:10.3389/fgene.2020.550515)
Supplement: Supplementary file 1 [file Table_1.DOCX]

**Table S1a: The sequences of adaptors, primers and barcodes**

| Name | Sequence 5’-3’ |
| --- | --- |
| Adaptor1 | 5’-ACACTCTTTCCCTACACGACGCTCTTCCGATCT-3’  3’-CGAGAAGGCTAGA**NNNNNN**-5’ |
| Adaptor2 | 5’-GTGACTGGAGTTCAGACGTGTGCTCTTCCGATCT-3’  3’-CGAGAAGGCTAGA**NNNNNN**-5’ |
| Primer1 | 5’-AATGATACGGCGACCACCGAGATCTACACTCTTTCCCTACACGAC-3’ |
| Primer2 | 5’-GTGACTGGAGTTCAGACGTGT-3’ |
| Index primer | 5’-CAAGCAGAAGACGGCATACGAGA**NNNNNN**GTGACTGGAGTTCAGACGTGT-3’ |
| Barcodes for each sample | A1 (CATCGG), A2 (AGCTAG), B1 (AGCTTC), B3 (TCGATC), C1 (TTAGGC), C4 (TAGCAC) |

.

Table S1b: Primer sequences and PCR conditions for bisulfite sequencing PCR

| Gene | Sequence of primer | Length/bp | Annealing temperature/℃ |
| --- | --- | --- | --- |
| *CDH13* | F: 5’-GAATTGTTGGGGTTATTTTATT-3’  R:5’-CCAAAACCAATAACTTTACAAA-3’ | 395 | 58 |
| *CXCR1* | F: 5’- TGGGTCAAGTTCATATGTTTAGGC-3’  R:5’-CCATGTGATTGCTGAGACCTGT -3’ | 378 | 58 |
| *METTL13* | F: 5’-TTTTTGGATGGTTGTTGTTGATTAG -3’  R:5’-TAAAAAACCCCTCACACACTTTTT -3’ | 392 | 58 |
