# Supplementary material for: Genome-Wide DNA Methylation Analysis of Mammary Gland Tissues From Chinese Holstein Cows With Staphylococcus aureus Induced Mastitis
Source: Front Genet. 2020 Oct 19;11:550515. doi: 10.3389/fgene.2020.550515 (PMC7604493; doi:10.3389/fgene.2020.550515)
Supplement: Supplementary file 10 [file Data_Sheet_1.docx]

Supplementary material

**Table S1** The sequence of adaptors and primers for Methyl-RAD sequencing and Bisulfite sequencing PCR

**Table S2** The identified CCGG/CCWGG DNA methylation sites in Chinese Holstein

**Table S3** The identified differently CCGG/CCWGG methylation sites in Chinese Holstein

**Table S4** The distribution of identified different CCGG/CCWGG methylation sites and genes in chromosomes of Chinese Holstein

**Table S5** The identified differently CCGG/CCWGG methylated genes between SA and CL group

**Table S6** Significant GO terms enriched by differently CCGG/CCWGG methylated genes

**Table S7** Significant KEGG pathways enriched by differently CCGG/CCWGG methylated genes

**Table S8** Functional enrichment results for CCGG DEMGs

**Figure S1 Bisulfite sequencing PCR results of METTL13 gene.** Columns represent the CpG sites and rows represent the clones. Black and white points represent methylated and unmethylated CpG sites, respectively. The blank region indicate that the CpG site was not detected by BSP.
