# Supplementary figures and images for: Genome-Wide DNA Methylation Analysis of Mammary Gland Tissues From Chinese Holstein Cows With Staphylococcus aureus Induced Mastitis
Source: Front Genet. 2020 Oct 19;11:550515. doi: 10.3389/fgene.2020.550515 (PMC7604493; doi:10.3389/fgene.2020.550515)

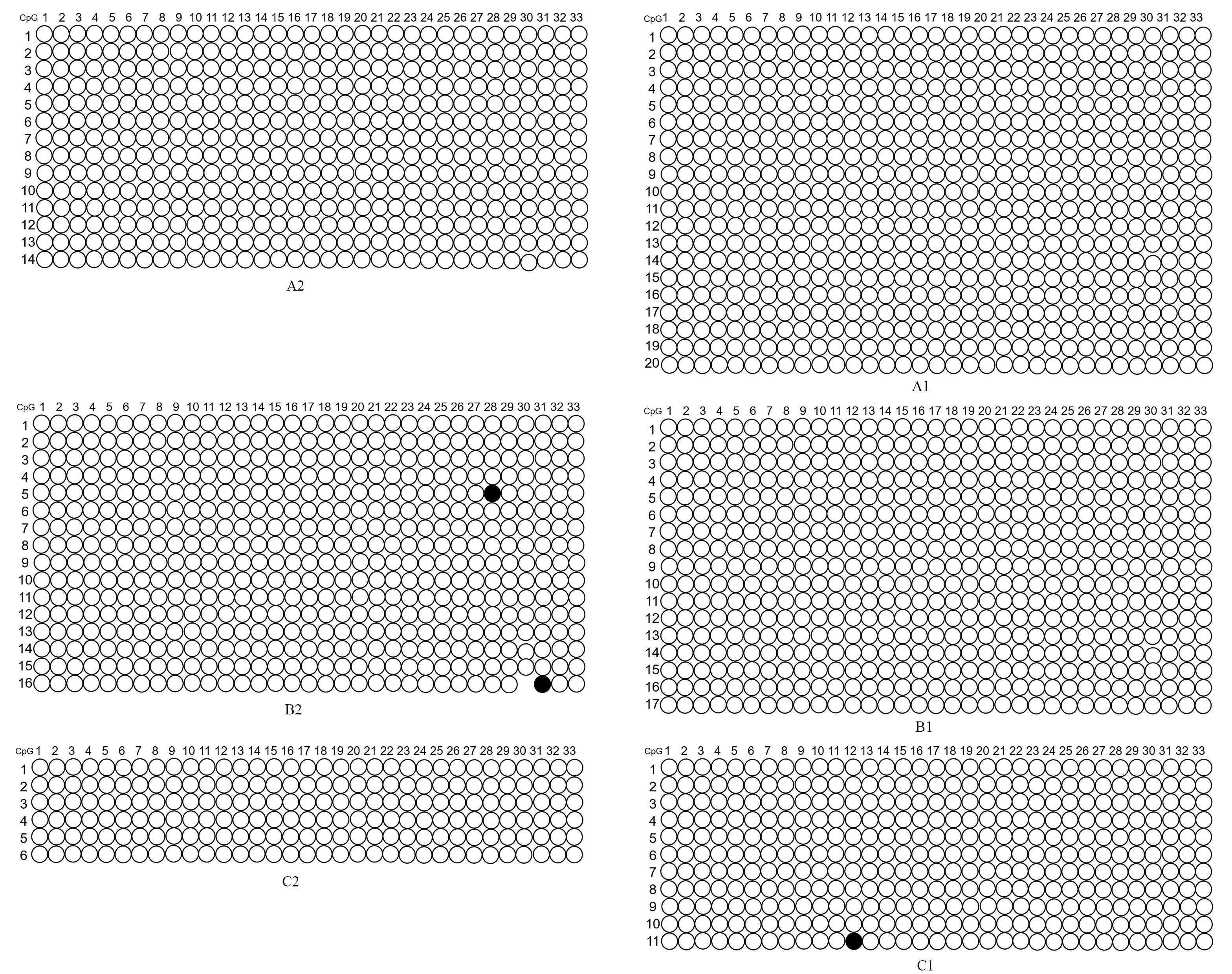

Supplement: Supplementary file 11 [file Image_1.TIF]
